# Stride-to-stride variability while backward counting among healthy young adults

**DOI:** 10.1186/1743-0003-2-26

**Published:** 2005-08-11

**Authors:** Olivier Beauchet, Véronique Dubost, François R Herrmann, Reto W Kressig

**Affiliations:** 1 Laboratory of Physiology and Physiopathology of Exercise and Handicap, Faculty of Medicine, University of Saint-Etienne, France; 2 Department of Geriatrics, Saint-Etienne University Hospitals, Saint-Etienne, France; 3 Department of Rehabilitation and Geriatrics, Geneva University Hospitals, Geneva, Switzerland

**Keywords:** Dual-task, Stride-to-stride variability, Attention, Gait control, Healthy young adults

## Abstract

**Background:**

Little information exists about the involvement of attention in the control of gait rhythmicity. Variability of both stride time and stride length is closely related to the control of the rhythmic stepping mechanism. We sought 1) to determine whether backward counting while walking could provoke significant gait changes in mean values and coefficients of variation of stride velocity, stride time and stride length among healthy young adults; and 2) to establish whether change in stride-to-stride variability could be related to dual-task related stride velocity change, attention, or both.

**Methods:**

Mean values and coefficients of variation of stride velocity, stride time and stride length were recorded using the Physilog^®^-system, at a self-selected walking speed in 49 healthy young adults (mean age 24.1 ± 2.8 years, women 49%) while walking alone and walking with simultaneous backward counting. Performance on backward counting was evaluated by recording the number of figures counted while sitting alone and while walking.

**Results:**

Compared with walking alone, a significant dual-task-related decrease was found for the mean values of stride velocity (*p *< 0.001), along with a small but significant increase for the mean values and coefficients of variation of stride time (*p *< 0.001 and *p *= 0.015, respectively). Stride length parameters did not change significantly between both walking conditions. Dual-task-related increase of coefficient of variation of stride time was explained by changing stride velocity and variability between subjects but not by backward counting. The number of figures counted while walking decreased significantly compared to backward counting alone. Further, the dual-task related decrease of the number of enumerated figures was significantly higher than the dual-task related decrease of stride velocity (*p *= 0.013).

**Conclusion:**

The observed performance-changes in gait and backward counting while dual tasking confirm that certain aspects of walking are attention-demanding in young adults. In the tested group of 49 young volunteers, dual tasking caused a small decrease in stride velocity and a slight increase in the stride-to-stride variability of stride time, while stride velocity variability was not affected by the attention-demanding task. The increase in stride time variability was apparently the result of a change in gait speed, but not a result of dual tasking. This suggests that young adults require minimal attention for the control of the rhythmic stepping mechanism while walking.

## Background

Dual-task related gait changes are usually interpreted as interference caused by competing demands for limited attentional resources [1], highlighting the idea that walking is not only an automatic process but also an attention-demanding task. For example, it has been shown that healthy young adults devote attention to the control of balance during single-limb support in an anxiety provoking condition [2]. The involvement of attention in the control of the walking-related rhythmic stepping mechanism remains less clear, with only a few and contradictory published results in the literature [3-7].

Stride time and stride length variability are both parameters that are related to the control of the rhythmic stepping mechanism [8]. In motor control in general, high variability is related to major attention involvement [9], whereas low variability reflects automatic processes that require minimal attention [9,10]. Performing a motor task while walking, such as carrying a cup [7] has been related to an increased variability of stride time, but not stride length. Verbal fluency task is a frequently used attention-demanding task in dual-task paradigm [1]. In contrast to attention-demanding motor tasks and to their older counterparts, young adults showed no significant change in stride-to-stride variability while performing a verbal fluency task [3,5,6]. Recently, Beauchet et al. [11] reported that, compared to a verbal fluency task, backward counting out loud from 50 significantly increased the coefficient of variation (CV) of stride time in a group of older adults aged 75 years and older who had a broad range of cognitive function abilities (e.g., some had mild dementia). The authors suggested that these findings could be explained by a possible age-related difficulty in the ability to appropriately allocate attention between both tasks due to a major competitive interaction with executive function while dual tasking. Little information is available about the impact of backward counting on stride-to-stride variability in healthy young adults. The only published study using this mental arithmetic task in a small group of healthy young adults showed that the CV of stride length while backward counting did not change compared with walking alone [12]. No data are available about the impact of backward counting on stride time variability.

Previous studies have shown that stride time variability increases when stride velocity decreases [13-16]. Because stride velocity often decreases under dual-task condition [1,12], dual-task related increase in CV of stride time could be provoked either by stride velocity decrease, the attention-demanding task, or both. The understanding of the role of stride velocity, as a potential confounder in the relationship between stride time variability and the involvement of attention in gait control is important. In contrast to stride time variability, variability of stride length in young adults remained low across different gait speeds while walking alone [17]. Furthermore, no significant stride length changes appeared under dual-task condition [3,5,6]. Such results suggest a constant stereotype pattern for stride length regulation, independent of gait speed.

We hypothesized that backward counting could provoke significant changes in stride time variability but not in stride length variability related to different attention involvement, independently of dual-task related changes in stride velocity among healthy young adults. The aim of this study was 1) to determine whether backward counting while walking could provoke significant gait changes regarding mean values and coefficients of variation of stride velocity, stride time and stride length among healthy young adults; and 2) to establish whether possible significant changes in stride-to-stride variability could be related to dual-task related stride velocity changes, backward counting, or both.

## Methods

### Participants

Forty-nine healthy young adults (25 men and 24 women, mean age 24.1 ± 2.8 years, range: 20–30 years) were recruited from the campus of Saint-Etienne University after having given their written informed consent. The young adults reported no physical and mental disorders. They took no medication. The study was approved by the local ethics committee and conducted in accordance with the ethical standards set forth in the declaration of Helsinki (1983).

### Tasks

The participants were asked to perform, in randomized order, the following tasks to the best of their capacity: counting backward aloud starting from 50 while sitting on a chair and while walking. For the dual-task condition, subjects were not specifically instructed to prioritize either one of both tasks, but were asked to perform the combined task at their best and at normal self-selected walking speed. Before testing, a trained evaluator gave standardized verbal instructions regarding the test procedure with visual demonstration of the walking test. To familiarize participants to the Physilog^®^-system [18,19], subjects completed 2 walking trials before recording. Participants' subjectively perceived gait safety while walking was measured with a visual analogue scale (score from 0 = safe to 10 = very unsafe) after each walking trial. All subjects reported zeroes under both conditions. Each subject completed one trial for each recorded walking condition. The subjects walked on a 20-meter walkway in a well-lit environment, at self-selected speed, and wearing their own footwear.

### Apparatus

Stride parameters were obtained using Physilog^® ^[18,19]. Physilog^® ^is a validated ambulatory gait analysis system based on miniature kinematic sensors (i.e. gyroscopes) attached on body segments and connected to a portable data logger worn at the waist. In this study, lower limb movement during walking was measured using 4 miniatures gyroscopes (Murata, ENC-03J) attached with a rubber band, respectively, to each shank and each thigh. After each walking trial, data were transferred from the data logger to a personal portable computer via an interface cable for analysis and storage. The temporal and spatial gait parameters were estimated from the angular velocity of the lower limbs. Gait phases were determined from the precise moments of heel-strike and toe-off. These events gave rise to distinctive features of the shank angular velocity signals in the form of rather negative peaks. An algorithm based on wavelet transformation was used to enhance the estimated times and, thus, to determine mean gait cycle duration (i.e., stride time). Mean stride length was calculated on the basis of double-segment gait model involving both shank and thigh. Mean stride velocity was defined as the average of all strides' instantaneous walking speeds, calculated from mean stride length and mean stride time.

### Study variables and outcomes

Stride velocity, stride time and stride length were measured during walking on a 20-meter walkway with Physilog^® ^[18,19]. To assure that gait parameters were collected while steady state walking, the first and last 2.5 meters corresponding to the acceleration and deceleration phase of each trial were excluded from analysis. The enumerated figures (i.e., subtractions of one) and errors of subtractions were recorded with a tape recorder. We defined the number of enumerated figures while walking as the number achieved during the time interval needed to walk over the 15 meters distance. The corresponding number at rest was defined as the number of figures that participants enumerated during the same time interval while sitting on a chair.

The following outcomes were used: 1) mean and standard deviation of mean values of stride velocity, stride time, stride length and number of enumerated figures under single and dual-task condition; 2) mean and standard deviation of CV (CV = ([standard deviation/mean] × 100) of stride velocity, stride time and stride length; and 3) normalized dual-task-related variation of gait speed and counting performance expressed as mean and standard deviation of dual-task-related mean value changes in stride velocity and number of enumerated figures under dual-task condition, calculated with following formula: 

### Statistical analysis

Main outcome measures such as stride velocity, stride time and stride length were summarized using means and standard deviations. The normality of the parameters' distribution was verified with a skewness and kurtosis tests before and after applying usual transformations to normalize non-Gaussian variables by taking the logarithmic transformation. First, all comparisons of the main outcome measures were performed with paired samples *t*-test. Second, two balanced analysis of covariance (*ANCOVA*) with a repeated measures design was performed, once for mean stride time and a second time for CV of stride time, to estimate the effects of counting backward, stride velocity and subjects (corresponding to the variability between subjects) without interaction terms, while adjusting for walking speed. For computing the error term, subjects were nested within walking conditions. *P *< 0.05 was considered statistically significant. All statistics were performed using the Stata Statistical Software 2003.

## Results

**Figure 1:**
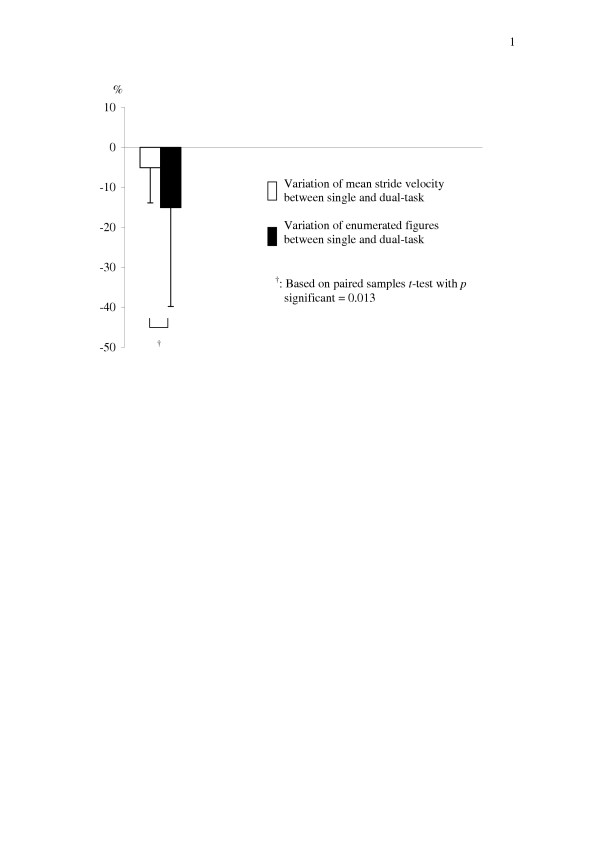
Change* in mean value of stride velocity and enumerated figures from single to dual-task condition among healthy young adults (n = 49). Error bars reflect the standard deviation. *: Calculated from the normalized difference between walking alone and walking with counting backward, i.e.

As shown in Table 1, dual-task related decrease in mean value of stride velocity was significant compared to walking alone (*p *< 0.001), whereas the CV of stride velocity did not change significantly (*p *= 0.097). Both mean value and CV of stride time were significantly higher while backward counting compared to walking alone (respectively, *p *< 0.001 for mean value and *p *= 0.015 for CV). No significant dual-task related changes in mean value and CV of stride length were found compared to walking alone (*p *= 0.414 and *p *= 0.275). Furthermore, significantly fewer figures were enumerated under dual-task than under single-task condition (*p *< 0.001). All subjects performed the mental arithmetic task without errors of subtractions. The *ANCOVA *models (Tables 2 and 3) revealed that both stride time parameters were significantly associated with walking speed and subject's effect (*p *< 0.010) but not with the simultaneous task of backward counting (*p *= 0.227 for mean value and *p *= 0.330 for CV). Moreover, R-squared values showed that the variance explained by the *ANCOVA *models was high for the mean value and CV of stride time (respectively 0.98 and 0.83). As the interaction term between task and velocity was neither significant for means of stride time nor for CV of stride time, we only reported the models without interaction. As depicted in Figure 1, the number of enumerated figures showed a higher decrease from walking alone to walking with backward counting than the dual-task related decrease in stride velocity (*p *= 0.013).

**Table 1:** Mean values and standard deviations of gait and backward counting parameters under single and dual-task condition among healthy young adults (n = 49)

	Single task	Dual-task	P-value*
Stride velocity			
Mean value (cm/sec)	129.7 ± 13.5	122.9 ± 16.0	<0.001
CV (%)	4.3 ± 2.0	4.7 ± 1.1	0.097
Stride time			
Mean value (ms)	1066.9 ± 81.7	1129.5 ± 138.1	<0.001
CV (%)	1.8 ± 0.8	2.1 ± 1.1	0.015
Stride length			
Mean value (cm)	137.4 ± 11.6	136.9 ± 11.7	0.414
CV (%)	3.9 ± 1.0	4.1 ± 1.0	0.275
Number of enumerated figures	18.9 ± 5.1	16.1 ± 3.8	<0.001

±: Standard deviation

CV: Coefficient of Variation = ([standard deviation/mean] × 100)

*: Compared to single task and based on paired samples *t*-test and use of normalized value by taking the logarithmic transformation

Sec: Second

**Table 2:** F test and *P*-value of *ANCOVA *with a repeated measures (n = 98) design comparing mean value of stride time while walking at self-selected speed with and without backward counting, adjusted for walking speed (covariate) and subject effect (n = 49).

**Source of variation**	**Sum of square**	**df**^||^	**Mean square**	**F**	**P-value**
Backward counting*	0.001	1	0.0008	1.50	0.227 †
Subjects ‡	0.431	48	0.0090	17.71	0.000
Log (Stride velocity) §	0.124	1	0.1242	244.80	0.000
Residual	0.024	47	0.0005		
Total	1.008	97	0.0104		

*: Backward counting coded as a binary variable (0 = walking alone, 1 = walking with backward counting), †: Box conservative estimate, ‡: Variability between subjects §: Normalized by taking the logarithmic transformation, ||: Degree of freedom

**Table 3:** F test and *P*-value of *ANCOVA *with a repeated measures (n = 98) design comparing coefficient of variation of stride time while walking at self-selected speed with and without backward counting, adjusted for walking speed (covariate) and subject effect (n = 49).

**Source of variation**	**Sum of square**	**df**^||^	**Mean square**	**F**	**P-value**
Backward counting*	0.060	1	0.0601	0.97	0.330 †
Subjects‡	12.751	48	0.2656	4.28	0.000
Log (Stride velocity) §	0.464	1	0.4636	7.47	0.009
Residual	2.915	47	0.0620		
Total	17.201	97	0.1773		

*: Backward counting coded as a binary variable (0 = walking alone, 1 = walking with backward counting), †: Box conservative estimate, ‡: Variability between subjects §: Normalized by taking the logarithmic transformation, ||: Degree of freedom

## Discussion

Our results show that, among a sample of healthy young University students, backward counting while walking provoked significant changes in gait and counting performance, with a greater dual-task effect on backward counting than on gait. The decrease in mean value of stride velocity under dual-task was solely related to the increase of mean value of stride time. Mean value and CV of stride length did not change during walking with simultaneous backward counting. Increased stride time variability in dual-task condition was explained by slower stride velocity and subjects' effect, but was not directly attributable to dual tasking. Further, the number of enumerated figures while walking decreased significantly.

Changes in gait patterns due to the simultaneous performance of a walking-associated task have been reported previously among healthy young adults and interpreted as interference related to competing demands for attention resources involved in both tasks [1]. Both dual-task-related performance changes in gait and backward counting found in our study support this statement. However, unlike previous results obtained in older adults that showed major dual-task related gait changes [1,11], only minor changes in gait parameters were found in our sample of healthy young adults. Several interpretations of these results are possible.

The explanation of dual-task interference is usually based on the assumption that attention resources are limited [20]. According to this theoretical approach, dual-task interference will only occur if the available central resource capacity is exceeded, provoking a performance decrease in one or both tasks. Therefore, interference suggests an overload of the central resources associated with an inability to appropriately adapt allocation of attention between two simultaneously performed tasks. The manner in which attention is divided between two tasks in dual-task paradigm mainly depends on both the priority given (or not) to one task and the attentional load of each task [1,20-22]. In our study, subjects were asked to combine both walking and backward counting without prioritizing either one of the tasks, creating a condition in which attention is divided. Both tasks used in our dual-task paradigm are relatively easy and do not require major attention. Backward counting out loud from 50 is a simple mental arithmetic task requiring low attention involvement in healthy young University students. Therefore, the total attentional load mobilized to simultaneously perform both tasks could not overload the available central resources, and thus only provoked little interference with minor gait changes. All significant dual-task related gait parameter changes in our study were relatively small. Gait speed decreased from 130 cm·s^-1 ^to 123 cm·s^-1 ^and the CV of stride time increased from 1.8 to 2.1%. In addition, although decrease of stride velocity while backward counting was related to increase of stride time, change in stride-to-stride variability for stride time was not associated with the attentional component of backward counting.

Previous studies have shown that dual-task related gait changes also depend on the type of measured stride parameters [1,7,8]. A change in single support time while performing a walking-associated attention-demanding task has been shown in healthy young adults [2], suggesting that young adults devote attentional resources to balance control during single-limb support. Few studies have explored the effect of a walking-associated task on the rhythmic stepping mechanism in young adults [4,6,7]. Our findings showed no significant effects of backward counting on means and CV of stride length. Furthermore, dual-task related changes in stride time could be explained by a decrease in stride velocity and variability between subjects, but apparently not on attentional components related to backward counting. Such results suggest that, in contrast to stride velocity, the control of the rhythmic stepping mechanism requires only minimal attention. Only two studies using a motor task as attention-demanding task while walking have shown significant modifications in stride time variability of young adults. Grabiner et al [7] found an increase in stride time variability while simultaneously carrying an 8-ounce cup placed in a saucer while walking. Ebersbach et al. [4] reported a significant decrease in stride time when walking with a rhythmic finger tapping task, interpreted as a magnet effect, a term used to describe the tendency of biological oscillators to attract each other. However, both studies did not examine the role of walking speed as a potential confounder in the relationship between stride time variability and the involvement of attention in gait rhythmicity control.

Most studies exploring dual-task related gait changes have focused on mean values of stride parameters [1], whereas stride-to-stride variability is considered as a sensitive marker for gait control [9,23,24]. Among the temporal gait parameters, stride time reflects the walking rhythm, and is therefore taken as an index of the rhythmic stepping mechanism control [9]. In older people, there is increasing evidence that stride time variability may be related to executive function. Recently, Hausdorff et al. [25] showed an association between high CV of stride time and a relative decline in executive function among healthy older adults, and Sheridan et al. [26] reported a similar relationship between high CV of stride time and impaired executive function in demented older adults. Furthermore, Beauchet et al. [11] recently reported a specific increase of CV of stride time in a group of older adults with a range of cognitive function abilities while backward counting, but not with a verbal fluency task. Whereas verbal fluency mainly relies on semantic memory [27], counting backward essentially depends on the working memory [28] and is therefore more directly related to executive functions. Thus, the dual-task-related increase in CV of stride time while counting backward could be related to competitive interaction with executive function.

The findings of the present study demonstrate that the dual-task related increase in mean value and CV of stride time was apparently related to stride velocity and subjects' effect, but not independently to attentional interference. Although the effect of stride velocity on variability is complex [29], similar positive correlations between increase in stride time variability and decrease of stride velocity have been reported previously [13-16]. Thus, it seems that in young adults the control of gait rhythmicity, stride velocity variability, stride length variability and likely stride time variability, is an automated process that demands little or no attention.

Interestingly, the decrease in the stride velocity during dual tasking was related to an increase in stride time but not to changes in stride length. This result confirms previous findings, which suggested that stride length is not affected by dual tasking, despite changes in gait speed and the performance of attention-demanding tasks [3,5,6,17]. Our subjects decreased stride velocity only by increasing their stride time, without modifying their stride length. This increase in stride time has been related to an increase in the double-support phase [1,2], which may serve to reduce attentional demands during the swing phase and lower the risk of a loss of balance under dual-task. Therefore, the change in the gait pattern during dual task might represent a strategy aimed at maintaining an optimal index of movement consistency in term of energy costs, attentional demand, and efficiency of gait control. The isolated increase in stride time under dual tasking may be explained by two interpretations. First, stride length and stride time could depend on different cerebral control areas. Second, stride time could be more sensitive to interference than stride length.

In our sample of young University students dual tasking had a greater effect on the performance of backward counting than it did on gait velocity. This result could be interpreted as an implicit strategy of the participants, in this specific dual-task situation, to rather give priority to gait safety than to arithmetic task performance. A similar strategy has been showed in older adults [30].

A possible methodological limitation of the present study might be related to the number of strides required to obtain a representative and suitable measure of stride-to-stride variability. Analyzing steady-state walking over 15 meters, the number of steps collected in our study was around 20, whereas Owings et al. claimed that accurate estimation of step kinematics variability required at least 400 steps [31]. Another question that calls for future study is how other, more difficult "dual-tasks" might affect the variability of gait in healthy young adults.

In conclusion, performance changes in gait and backward counting when both tasks are performed simultaneously confirm that walking is an attention-demanding task in young adults. Backward counting caused a small, but significant decrease in stride velocity. However, this dual-task did not affect stride length variability and the small change in stride time variability was apparently related to the change in mean stride velocity Apparently, young adults do not allocate much attention to the control of the rhythmic stepping mechanism of walking.

## Conflict of interest statement

The author(s) declare that they have no competing interests.

## Contributors

O Beauchet was the main investigator of the study, designed the study, participated in data analysis, and wrote the manuscript. V Dubost was responsible for data collection and participated in preparation and analyses of data, and writing of the manuscript. FR. Herrmann participated in the development of statistical analysis, analysis, and writing of the manuscript. RW Kressig participated in the development of statistical analysis, data analysis, and writing of the manuscript.
